# Moving beyond abundance distributions: neutral theory and spatial patterns in a tropical forest

**DOI:** 10.1098/rspb.2014.1657

**Published:** 2015-03-07

**Authors:** Felix May, Andreas Huth, Thorsten Wiegand

**Affiliations:** Department of Ecological Modelling, Helmholtz-Centre for Environmental Research—UFZ, Permoserstraße 15, Leipzig 04318, Germany

**Keywords:** beta-diversity, distance decay, pattern-oriented modelling, point-pattern analysis, spatially explicit neutral model, species–area relationship

## Abstract

Assessing the relative importance of different processes that determine the spatial distribution of species and the dynamics in highly diverse plant communities remains a challenging question in ecology. Previous modelling approaches often focused on single aggregated forest diversity patterns that convey limited information on the underlying dynamic processes. Here, we use recent advances in inference for stochastic simulation models to evaluate the ability of a spatially explicit and spatially continuous neutral model to quantitatively predict six spatial and non-spatial patterns observed at the 50 ha tropical forest plot on Barro Colorado Island, Panama. The patterns capture different aspects of forest dynamics and biodiversity structure, such as annual mortality rate, species richness, species abundance distribution, beta-diversity and the species–area relationship (SAR). The model correctly predicted each pattern independently and up to five patterns simultaneously. However, the model was unable to match the SAR and beta-diversity simultaneously. Our study moves previous theory towards a dynamic spatial theory of biodiversity and demonstrates the value of spatial data to identify ecological processes. This opens up new avenues to evaluate the consequences of additional process for community assembly and dynamics.

## 1. Introduction

A fundamental goal of ecology is to understand the mechanisms that determine the spatial distribution of species and the dynamics of communities [1,2]. Plant diversity of wet tropical forests has been especially challenging for species coexistence theories [3] and their complexity has so far hindered a comprehensive understanding of the relative importance of the processes that govern the spatial structure, the composition and dynamics of these systems [4,5].

While classical coexistence theories have focused on niche differences among species [6], neutral theory has used the opposite starting point and assumes that all species share equal *per capita* fitness [7]. The neutral model introduced by Hubbell [7] is not spatially explicit, i.e. the positions of individuals are not specified and, therefore, this model can only provide non-spatial predictions. A series of key studies of neutral theory focused on explaining one fundamental diversity pattern: the species abundance distribution (SAD), e.g. [8,9]. Neutral theory initiated a controversial debate in community ecology, and it has been ardently discussed if neutral models provide a better fit to observed SADs than previous (statistical) models [8,10,11]. However, due to this restricted focus on SADs, the debate has not resulted in clear progress but rather highlighted the fundamental challenge of equifinality, which means that several contrasting models, including neutral as well as niche-based approaches, can fit patterns such as the SAD equally well [9,12].

More recent neutral models were spatially explicit, i.e. each individual was characterized by its position in space [13–15]. Applications of these models demonstrated that neutral theory is also able to reproduce realistic beta-diversity patterns [16] and realistic species–area relationships (SARs) [14]. However, focusing on single biodiversity patterns is unlikely to move community ecology forward, because a single pattern provides only limited information and thus little power to discriminate between contrasting models and theories [12,17]. Instead, there is a need for simultaneous comparisons of a wide array of predictions of a theory with field observations, as outlined by the concepts of strong inference [18] or pattern-oriented modelling [19,20].

To overcome this obstacle, we here apply a general framework of model–data integration to advance our understanding of the dynamics of species-rich plant communities at local spatial scales. More formally, our approach involves comparisons of model predictions and field observations using data aggregations that quantify key forest attributes, such as the SAD or the SAR. They are also called ‘patterns’ within the framework of ‘pattern-oriented modelling’ [20] and are known as ‘summary statistics’ in the framework of approximate Bayesian computation [21,22]. To avoid the problem of equifinality, our approach uses multiple patterns and we require the model to correctly predict several of the observed patterns simultaneously [20]. Each pattern can be considered as a ‘filter’ that helps to identify unrealistic parameter sets or model structures [23].

We argue here that suitable diversity patterns are provided by the spatially explicit positions of individual trees in a forest. Such information is now provided by forest dynamics plots, where the sizes and locations of all trees are monitored and censused every 5 years at plots of up to 50 ha [24]. Confronting dynamic models with spatial patterns observed in plant communities is also a logical next step as most mechanisms suggested to promote coexistence in species-rich communities are assumed to have a strong spatial component [25]. Studies that analysed forest structure with spatial point-pattern statistics [26] provided new insights into the spatial structure of forest communities [27–31]. However, detailed spatial data have been rarely used for the parametrization and validation of dynamic, process-based simulation models, but see e.g. [32].

In order to link spatially explicit field observations with model predictions, we require a spatially explicit model of community dynamics that produces output data of the same structure as the census data of the forest dynamics plots. To closely tie such a model to existing theory, we developed and analysed a novel spatially explicit extension of the neutral model of Hubbell [7], called CONFETTI. In contrast to previous spatially explicit neutral models that were usually implemented on a two-dimensional regular lattice, where each individual occupies one discrete grid cell, e.g. [13,15], in our model each individual in the local community has an explicit location in continuous space [33]. This allows for a more detailed representation of local competition and facilitates a direct comparison of model predictions with the data of forest plots. Recent advances in statistical inference for stochastic simulation models [21,22] allow now for rigorous model parametrization of stochastic simulation models based on multiple observed patterns.

In this study, we confront predictions of our spatial neutral model with data from the 50 ha forest plot at Barro Colorado Island (BCI), Panama. For this purpose, we calculate several non-spatial and spatial summary statistics of the BCI plot, each of which capture a different key aspect of forest dynamics and spatial biodiversity structure. The patterns include the mortality rate, the species richness (SR), the SAD (non-spatial patterns), and the pair-correlation function, the SAR and beta-diversity (spatial patterns). We use the model and data to answer the following specific questions: (i) can our spatially explicit neutral model provide realistic predictions for each pattern independently? (ii) Can several patterns be predicted at the same time, i.e. with the same parameter set, with the neutral model? If yes, which ones? (iii) How do the answers to (i) and (ii) depend on the uncertainty assigned to each pattern?

We show that the neutral model is able to fit each pattern individually with a precision similar to the observed variation during the 20-year census period; that the model correctly reproduces several non-spatial and spatial patterns at the same time; but that it is unable to predict the SAR and beta-diversity simultaneously. This suggests that these two patterns convey important information on the underlying dynamic processes. The framework for model–data integration presented here provides new avenues for assessing the roles of additional processes, e.g. negative conspecific density, on community assembly and dynamics.

## 2. Material and methods

### (a) Study area

The tropical forest at Barro Colorado Island (BCI), Panama (9°10′ N, 79°51′ W) is a seasonally moist tropical forest that hosts more than 300 tree and shrub species. Rainfall averages 2600 mm per year, with a pronounced dry season. Investigations were carried out within the 50 ha forest dynamics plot, which consists of mainly old growth lowland moist forest. Elevation ranges from 120 to 155 m.a.s.l. The plot was established in 1982 and all trees with diameter at breast height (dbh) ≥1 cm have been mapped, tagged and measured every 5 years since 1985 [24,34,35].

### (b) The spatial neutral model

To closely tie our approach to existing theory and to spatial data from forest plots, we developed a spatially explicit extension of the spatially implicit neutral model of Hubbell [7] called CONFETTI. Our model differs from previous grid-based neutral models [13–15] in two important ways. First, we maintained the distinction between a metacommunity and a local community introduced by Hubbell [7] and second, the model is spatially continuous. Owing to this novel model design, our model predictions are directly comparable with predictions of the classical spatially implicit neutral model and allow a direct comparison with spatially continuous forest census data using point-pattern analysis.

The model CONFETTI simulates stochastic survival, mortality, recruitment and immigration of all adult trees in the local community following the assumptions of *per capita* neutrality and of zero-sum dynamics. In the following, we provide a brief introduction of the model, while a complete model description is provided in the electronic supplementary material, appendix S1 and figure S1.

In the model, each tree is characterized by its location in space and its species identity. We simulated 21 100 tree individuals in a spatial arena of 50 ha (1000 × 500 m). This number of trees corresponds to the average number of trees with dbh ≥ 10 cm in the 50 ha BCI plot. Competition among trees is modelled based on the zone-of-influence (ZOI) approach [36], where trees interact with other trees only within a circular ZOI around their central point. To guarantee *per capita* neutrality, all trees are assumed to have the same ZOI radius (*r*_t_). The survival probability of a tree decreases with the competition experienced by that tree. The competition process in the model requires three parameters: the ZOI radius (*r*_t_), the survival probability in the absence of competition (*b*_s_) and the competition pressure that reduces survival probability by 50% (*a*_s_).

Newly recruited trees can either be offspring of trees in the local community or immigrants from the metacommunity [7]. For local recruitment, we randomly select a mother tree and determine the location of the new recruit based on a log-normal distance kernel [37]. The two parameters of the dispersal kernel are the mean *d*_m_ and the standard deviation *d*_sd_ of the distance between mother tree and offspring.

Immigration from the metacommunity takes place with probability *m* (immigration rate) [7]. However, the immigration rate is not an independent parameter, but we link the immigration rate to the mean recruitment distance as suggested by [38]: *m* = *Pd*_m_/(*πA*), where *P* is the perimeter of the plot (3000 m), *d*_m_ is the mean distance between mother tree and offspring and *A* is the plot area (50 ha).

The non-spatial metacommunity is simulated based on a sequential construction scheme [39], where the abundance distributions and thus the diversity of the metacommunity is determined by the fundamental biodiversity number *θ*, which is defined as *θ* = 2*J*_M_*ν*, where *J*_M_ is the number of individuals in the metacommunity and *ν* is the speciation rate by point mutation [7]. The model CONFETTI is implemented in C++ and the code is available from the authors upon request.

## 3. Linking model and data

### (a) Sampling of model parameters

In order to assess the ability of the model to approximate the field observations of the BCI plot, we randomly created 2 000 000 parameter sets based on uniform priors from biologically plausible parameter ranges (electronic supplementary material, table S1). With each parameter set, we simulated forest dynamics for 100 generations (*ca* 5000 years) which was sufficient to reach a dynamic equilibrium (electronic supplementary material figure, S2). From each BCI census and the last step of each model simulation, we then calculated several summary statistics to quantify non-spatial and spatial patterns in an analogous way.

### (b) Non-spatial and spatial patterns

As non-spatial patterns we used the annual mortality rate (mort) [40], the SR and the SAD [7,8]. As summary statistics of spatial structure, we estimated the pair-correlation function of all trees *g*(*r*), the proportion of conspecific neighbours *F*(*r*) at distance *r* and the species–area relationship SAR(*r*). These three patterns were selected to represent contrasting aspects of spatial forest structure.

The pair-correlation function *g*(*r*) is the expected density of trees within distance *r* of a randomly selected tree, divided by the overall density λ of trees in the plot and quantifies the aggregation of all large trees irrespective of species identity [26]. Values of *g*(*r*) < 1 indicate hyper-dispersion at distance *r* and values of *g*(*r*) > 1 aggregation.

As a measure of beta-diversity, we used the proportion of conspecific neighbours *F*(*r*), which quantifies species mingling versus intraspecific aggregation. *F*(*r*) estimates the probability that two randomly chosen trees at distance *r* belong to the same species [16]. For independent and completely random species patterns we find *F*(*r*) = 1 − *D*, where *D* is the classical Simpson diversity index. Values of *F*(*r*) >> 1 − *D* indicate strong intraspecific aggregation, whereas *F*(*r*) < 1 − *D* indicates species mingling [16,26]. The function *F*(*r*) is also known as distance decay of community similarity [41].

Within the point-pattern framework, the SAR quantifies the expected number of species within a circular area *A* = *πr*^2^ with radius *r* [26, §3.1.5.2]. Both, *F*(*r*) and SAR(*r*) consider the position and species identity of all trees. However, the SAR(*r*) is much more influenced by rare species, while *F*(*r*) is largely dominated by the most abundant species [41].

We estimated the six summary statistics from the five BCI censuses between 1985 and 2005 using all living trees with dbh > 10 cm, as neutral theory only applies to adult trees [7]. Finally, we averaged the six summary statistics over the five censuses or the four inter-census periods, respectively.

In the model simulations, we derived the summary statistics from the tree community at the end of the simulation run after 100 tree generations. All spatial summary statistics were estimated for distances of 1–50 m, which includes the spatial scales where trees are assumed to compete for light and resources [42], and the typical scales of seed dispersal in BCI [43].

### (c) Comparison between simulated and observed summary statistics

For each parameter set *p*, we quantified the deviation between simulated (*S*_sim_) and observed summary statistics (*S*_obs_) by calculating the mean relative deviation mRD*_i_*(*p*) for each summary statistic *i* [21]3.1
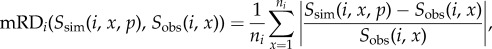
where *S*_obs_(*i*, *x*) is the average summary statistic *i* derived from the five BCI censuses, and the index *x* specifies the abundance class in the SADs or the radius *r* for the three spatial patterns. For the scalar summary statistics mortality rate and SR, we have *n*_i_ = 1, and we evaluated the three summary statistics of the spatial patterns at the distances *r* = 1, 2, 5, 10, 20 and 50 m (i.e. *n*_i_ = 6). A value mRD*_i_*(*p*) = 0 indicates perfect model–data fit, while increasing values indicate a decreasing goodness-of-fit [21].

### (d) Considering uncertainty in the observed data

To define the match versus mismatch of model predictions and data, we have to take the inherent variability of the data into account. To this end, we defined for each summary statistic *i*, a threshold for the deviation measure mRD*_i_* that reflects the variability in the corresponding observed pattern. We used the repeated censuses of the BCI plot to define a baseline level of uncertainty *ε*_*i*_ in pattern *i*, which represents the observed variation during the 20-year census period. We defined *ε*_*i*_ as the average mRD_*i*_ between the observed summary statistics for each single census and the average summary statistics over all censuses (table 1).

**Table 1.** Selection thresholds for all summary statistics used in the rejection sampling approach. The values of *ε*_*i*_ were calculated for each summary statistics as the average mean relative deviation equation (3.1) between the five BCI censuses (1985–2005) and the average summary statistics of these censuses. For each value of *f*, all possible 63 combinations out of the six summary statistics were used to select simulation results that match the observations at the respective error level. mort, annual mortality rate of trees (dbh ≥10 cm); SR, species richness at 50 ha; SAD, species abundance distribution; *g*(*r*), pair-correlation function; *F*(*r*), proportion of conspecific neighbours (beta-diversity); SAR(*r*), species–area relationship.

	mort	SR	SAD	*g*(*r*)	*F*(*r*)	SAR(*r*)
min (*f* × *ε*_i_, 0.2), with *f* = 1	0.068	0.013	0.089	0.007	0.029	0.012
min (*f* × *ε*_i_, 0.2), with *f* = 2	0.136	0.026	0.178	0.014	0.058	0.024
min (*f* × *ε*_i_, 0.2), with *f* = 5	0.200	0.065	0.200	0.035	0.145	0.060
min (*f* × *ε*_i_, 0.2), with *f* = 10	0.200	0.130	0.200	0.070	0.200	0.120

The variation between the BCI censuses includes only temporal variation, but in a statistical sense this variation occurs within the same ‘realization’ of the forest community dynamics. By contrast, model simulations—even with the same parameter set—represent completely independent realizations of community dynamics. Accordingly, a fair comparison between model and data requires taking into account the—unfortunately unknown—total uncertainty in observed forest patterns. To allow for larger levels of uncertainty, we introduce a tolerance factor *f* that describes how much larger the level of uncertainty in the summary statistics would be relative to the minimal uncertainty described by *ε*_*i*_. Thus, for mean relative deviations mRD_*i*_ < *f* × *ε*_*i*_, we accept the parameter set *p* for pattern *i* because in this case the observed and simulated patterns cannot be distinguished given the assigned level *f* of uncertainty (table 1).

### (e) Model parametrization

We applied a rejection sampling approach to select parameter sets that produced agreement between model simulations and field observations [22,23]. Specifically, we applied the selection criterion mRD_*i*_(*p*) < min(*f* × *ε*_*i*_, 0.2) to accept a parameter set only if the predicted mean relative deviation for summary statistic *i* was smaller than the uncertainty in the observed summary statistic (*ε*_*i*_) multiplied with tolerance factor *f*.

To answer our first question (i.e. whether neutral dynamics provide realistic predictions for each pattern independently), we applied the rejection sampling approach for each summary statistic independently and set *f* = 1, which means the model predictions had to match observations at the uncertainty level of the data. To answer our second and third question (i.e. whether our simple model can predict several patterns simultaneously and how this depends on the level of uncertainty), we considered all 63 possible combinations of patterns and varied the tolerance factor *f* (table 1). For each of these combinations we count the number of parameter sets that fulfil the rejection criterion and estimate the approximate posterior distribution of each parameter. To exclude parameter sets with poor match, we always limit *f* × *ε*_i_ < 0.2, thus we expect in any case that simulated and observed summary statistics deviate on maximum by 20%.

## 4. Results

### (a) Predictions of single patterns

**Figure 1.**
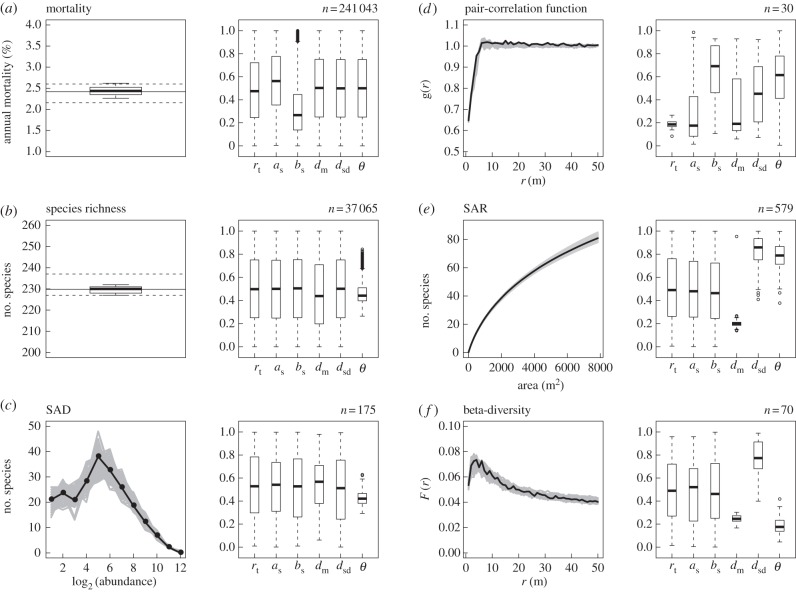
Model predictions and field observations when parametrizations were selected for each pattern independently. For each pattern *i*, the selection criterion was mRD_i_ < min(1 × *ε*_i_, 0.2) (table 1). The panels on the left show simulation results and observations, and the boxplots on the right summarize the selected parameter values. For comparability the parameters were standardized to the range [0;1] (see electronic supplementary material Table S1). The number of selected parameter sets (*n*) is provided in each row. For the scalar patterns—(*a*) mortality and (*b*) species richness—the boxplots on the left summarize the *n* simulation results, while the solid line and the dashed horizontal lines indicate the mean and the range in the five BCI censuses. For the SAD (*c*) and the spatial patterns (*d*–*f*), the grey lines show the *n* simulation results and the black solid lines show the observed pattern averaged over the five BCI censuses.

We found that our model is able to match each single pattern independently (figure 1). The number of accepted parameter sets, which provides a measure of the information contents of the respective pattern [19], varies largely among the six patterns. While the two scalar patterns—mortality rate and SR—can be fitted by a high proportion of the parameter sets, fits of the abundance distribution (SAD) or the spatial patterns [*g*(*r*), SAR(*r*), *F*(*r*)] were only possible with a limited number of parameter sets (figure 1).

Each pattern restricted the posterior parameter ranges in a different way, i.e. different patterns were informative on different processes and parameters. Fitting the SR or the SAD primarily restricted the fundamental biodiversity number *θ* of the metacommunity (figure 1*b*,*c*). Considering the spatial patterns, the pair-correlation function *g*(*r*) restricted the ZOI radius *r*_t_ (figure. 1*d*), while both, the SAR(*r*) and beta-diversity *F*(*r*) restricted *θ*, the mean and the standard deviation of the recruitment distance *d*_m_ and *d*_sd_ (figure 1*e* and *f*).

The question remains, whether patterns that constrain the same parameters result in compatible or in contrasting parameter estimates. For example, the range of *θ* required to fit the SAD: *θ* = 44.3–51.9 (25–75% quantile) was compatible with the range that fits the SR: *θ* = 45.8–55.8 (figure 1*b*,*c*). By contrast, fitting the SAR required higher values of *θ* = 74.2–88.0 (figure 1*e*), while fits of beta-diversity required low values of *θ* = 22.4–30.8 (figure 1*f*). Notably, the parameter ranges that produce good fits of the SAR and the beta-diversity agree for the recruitment kernel parameters (i.e. *d*_m_ and *d*_sd_), but are clearly incompatible for *θ* (figure 1*e* and *f*). This indicates that it might be impossible to fit the SAD, the SAR(*r*) and *F*(*r*) with the current model simultaneously, however, this may only be the case on the lowest level of uncertainty (i.e. *f* = 1).

### (b) Simultaneous match of several patterns

From the definition of the six patterns used here, we could distinguish summary statistics that depend on the species identity of trees [SR, SAD, *F*(*r*), SAR(*r*)] and others that do not [mort, *g*(*r*)]. As competition and survival are modelled independently of species identities, we expect that the model parameters related to competition and survival (*r*_t_, *a*_s_, *b*_s_) should primarily influence the summary statistics mortality rate and the pair-correlation function *g*(*r*). Indeed, at the tolerance level of *f* = 2, we found that filtering the simulation results with mortality and *g*(*r*) strongly constrained the range of the ZOI radius (*r*_t_) and the survival parameters (*a*_s_,*b*_s_) (electronic supplementary material, figure S3).

An interesting question is, if the joint information contained in the three non-spatial patterns is also able to constrain the spatial patterns. At the tolerance level of *f* = 2, we found 2375 parameter sets which correctly predicted the three non-spatial patterns (mort, SR and SAD) at the same time (electronic supplementary material, figure S4*a*–*c*). However, the information in the non-spatial patterns was clearly not sufficient to constrain the parameter space to predict reasonable spatial patterns (electronic supplementary material, figure S4*e*,*f*,*g*).

**Figure 2.**
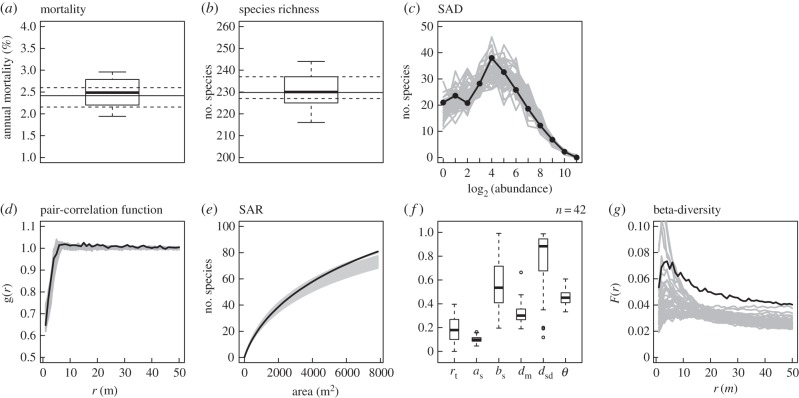
Same as figure 1, but here we required simultaneous match of all patterns except beta-diversity at the level of uncertainty of *f* = 5 (table 1).

When requiring simultaneous fit of all patterns except beta-diversity, we found 42 parameter sets at the tolerance level of *f* = 5 that yielded simultaneous realistic predictions (figure 2). All posterior parameter ranges were now relatively narrow, especially for *a*_s_, *d*_m_ and *θ* (figure 2*f*). However, in this case the predicted beta-diversity was generally too low, especially at larger distances *r* (figure 2*g*), which means the model predicted lower aggregation and higher mingling of species than observed in BCI.

Only at the highest tolerance level investigated here (*f* = 10), the model predictions matched all six patterns simultaneously (electronic supplementary material, figure S5). However, in this case there was a clear underestimation of the SAR at large spatial scales (electronic supplementary material, figure S5*e*).

## 5. Discussion

Our findings advance the debate about neutral theory by confronting a spatially explicit and spatially continuous neutral model with a comprehensive set of spatial and non-spatial forest attributes derived from census data of a tropical forest. Such an approach was impossible a few years ago, but recent advances in individual-based modelling [44], statistical inference for stochastic simulation models [21,22] and high performance computing allowed now for this new and refreshing approach on neutral theory in particular and plant community dynamics in general.

We found that our spatially explicit extension of the classical neutral model was able to fit all patterns individually with a precision at the level of uncertainty inherent to the data. We also found that the model could fit up to five of the six selected patterns simultaneously on a relatively narrow level of tolerated uncertainty. This means that already a simple model incorporates enough mechanisms to correctly predict several fundamental patterns of the diverse BCI forest. However, the failure of simple models can be informative, because mismatch between model predictions and field observations provides the basis for the subsequent identification of important ecological processes [19,20,45]. The failure of the spatially explicit neutral model to simultaneously predict the observed SAR and the beta-diversity (distance decay of similarity) suggests that these two patterns, when considered simultaneously, contain important information on the underlying ecological processes [41]. The neutral model underestimated the distance decay when fitting the other patterns well (figure 2*g*). It has been shown before that abundant species have the strongest influence on the distance decay [41]. Therefore, the mismatch between observed and modelled beta-diversity might indicate that the model underestimates the aggregation of abundant species and/or overestimates the co-occurrence of abundant species. In ‘The role of additional processes for tropical forest dynamics’, we suggest additional processes, such as species-specific dispersal and habitat associations, which are potentially important to predict both spatial patterns simultaneously. Thus, our understanding of forest dynamics can be improved by testing whether the extended models discussed below can fit an extended set of biodiversity patterns together.

### (a) Parameter estimates and model predictions

When we used only the SAD for parameter estimation, we obtained posterior parameter ranges for the fundamental biodiversity number *θ* and the immigration rate *m* that are in good agreement with previous fits based on the spatially implicit neutral model (*θ* ≈ 47–50, *m* ≈ 0.1–0.13) [8,11]. Note that the immigration rate was not independent, but defined by the mean recruitment distance (*d*_m_) as *m* = *P* × *d*_m_/(*π* × *A*) [38]*.* Thus, our spatially explicit neutral model agrees with the predictions of the spatially implicit model if we disregard its spatial output. This result is an important proof of concept for our model.

It is encouraging that our estimates for the mean recruitment distance (*d*_m_) were relatively similar to previous estimates based on seed trap data [16,43]. This is remarkable because the recruitment of adult trees in our model essentially integrates the processes of seed dispersal, seedling establishment, sapling growth and survival to adulthood into an effective recruitment distance. The similarity of our estimates for the mean recruitment distance with direct estimates of mean dispersal distances is reassuring and shows that our aggregated model captured the key process of dispersal limitation in a reasonable way.

### (b) The links between patterns and processes

We found that different patterns constrained distinct parameters, which indicates that there are specific links between patterns and processes. Specifically, patterns that did not depend on species identities—mortality and pair-correlation function—primarily constrained the parameters that describe competition. By contrast, the summary statistics that represent biodiversity patterns—SAD, SAR(*r*), *F*(*r*)—constrained the fundamental biodiversity number, as well as the recruitment kernel parameters. This clear distinction depends on the fact that in the neutral model competition does not depend on species identity and local species diversity. As soon as non-neutral differences among species are considered, this distinction between diversity independent and dependent patterns would be likely to break down.

The three spatial statistics used here are by no means exhaustive of the range of potential summary statistics provided by point-pattern analysis [26]. For example, the uni- and bivariate distribution patterns of species and species pairs [e.g. 27,28,46], and relationships between individual species and local SR [29] could be studied in future work. These summary statistics may prove informative of additional ecological processes.

### (c) The role of additional processes for tropical forest dynamics

Numerous studies have found strong evidence for non-neutral processes in tropical forests [4,47], albeit often for seedlings and saplings, which were purposefully excluded from the neutral model. However, while these analyses could document the occurrence of a mechanism, they cannot usually assess its relative importance for community dynamics and spatial structure, but see e.g. [48,49]. Statistical significance does not necessarily imply that the effect also has a major impact on the dynamics of the system [5]. The model–data integration approach presented here allows an assessment of the relative importance of ecological processes on key biodiversity patterns. While we limited our analysis here to neutral processes and found that the model cannot correctly predict the distance decay of similarity together with the other patterns, our model can be expanded to include species differences in a number of processes (i.e. including non-neutral processes) to identify the cause of this failure.

For example, spatially limited recruitment causes the aggregation of conspecific individuals in our model. Previous studies indicated that conspecific aggregation is a common pattern [27], and also that the degree of aggregation varies considerably among species [41,50]. These interspecific differences in conspecific aggregation can potentially resolve the incompatibilities in the simultaneous predictions of spatial diversity patterns. The most likely mechanism generating these differences in aggregation are species-specific differences in dispersal distances, due to different seed or fruit morphologies and different dispersal vectors [43,51].

Another process that has been often advocated to be an important driver of species diversity is negative conspecific density dependence (NDD), e.g. due to Janzen–Connell effects [4]. For tropical forests there is evidence that NDD plays an important role at the seedling stage [47,48,52], but there has been less support for NDD at the stage of adult trees [30,42]. Theoretical studies showed that there are clear differences in the spatial patterns predicted by neutral models compared with models incorporating negative conspecific density dependence [13,33], however, these models have not yet been linked to field observations.

Previous work found significant associations of species distributions to environmental covariates, such as terrain slope, elevation and soil nutrients at the spatial scales considered here, e.g. [53–55]. Other studies cast doubts on whether these habitat associations translate into substantial improvement in predicting spatial patterns [56]. However, habitat associations have a strong potential to constrain species distributions and thus also to influence the shape of spatial biodiversity patterns.

The three processes of interspecific variability in recruitment distances, negative density dependence and habitat associations can in principle be incorporated into the presented framework. Assessing the consequences of these processes is however beyond the scope of this paper and applying the approach presented here to investigate the implications of these additional processes will be computationally demanding.

## 6. Conclusion

Our study shows that the parametrization of spatial models of community dynamics requires the simultaneous use of different observed biodiversity patterns. Spatial data of fully mapped plots in particular can provide the additional information needed to distinguish among competing models that was missed in earlier approaches.

Fitting a spatially explicit extension of the classical neutral model to spatial data reveals why the debate around neutral models to data has not resulted in much progress. The neutral model is able to fit individual biodiversity patterns, such as the SAD and the SAR, with a precision at the level of the observed inter-census variability. We argue that progress in identifying important processes can only be achieved by assessing the simultaneous fit of several biodiversity patterns. Recent advances in inference for stochastic simulation models and the information in large fully mapped forest plots provide the methods as well as the data for implementing this research agenda.

## Supplementary Material

Supplement A

## Data accessibility

The census data for the BCI plot are publicly available at the website of the Center for Tropical Forest Science: http://ctfs.arnarb.harvard.edu/webatlas/datasets/bci/. Any use of the data has been agreed upon with the principal investigators of the BCI plot: Stephen Hubbell, Richard Condit and Robin Foster.
