# Supplementary material for: Moving beyond abundance distributions: neutral theory and spatial patterns in a tropical forest
Source: Proc Biol Sci. 2015 Mar 7;282(1802):20141657. doi: 10.1098/rspb.2014.1657 (PMC4344136; doi:10.1098/rspb.2014.1657)
Supplement: Supplement A [file rspb20141657supp1.pdf]

Supplementary Material for the article:

Moving beyond abundance distributions – neutral theory and spatial patterns in a tropical forest

Felix May (felix.may@ufz.de), Andreas Huth, Thorsten Wiegand

Appendix S1: Detailed model description of the spatially explicit neutral model CONFETTI

The model CONFETTI simulates stochastic survival, mortality, recruitment, and immigration of all adult trees in the local community following the assumptions of per-capita neutrality and of zero-sum dynamics. The name is simply derived from a snapshot of the model. If each tree is represented as a circle and the tree color indicates the species identity a plot of trees in the forest looks like confetti (see Fig. S1). In the following we present a complete description of the modelled processes, the simulation scheduling and the parameter ranges considered.

Competition

We modelled competition among trees using the zone-of-influence (ZOI) approach [1,2]. Accordingly, trees acquire resources and interact with other trees only within a circular zone-of-influence around their central point. We did not simulate tree size growth and assigned the same ZOI radius (r_t) to all trees due to the assumption of per-capita neutrality (Fig. S1). We defined a competition index for each tree by considering its overlap with all neighbouring trees:

$$CI_i = \frac{\sum_{j=1}^{N} A_{ij}}{A_i}, \qquad \text{Eq. S1}$$

where CI_i is the competition index of the focal tree i, N is the number of trees overlapping with the focal tree, A_{ij} is the overlapping area of the focal tree with the neighbour tree j and A_i is the ZOI area of the focal tree, which equals $\pi \times r_t^2$. We assume that the survival probability of a tree ($P_{surv}(i)$) decreases with competition. The competition index is related to the survival probability as an adapted Michaelis-Menten function:

$$P_{surv}(i) = b_s \times \left(1 - \frac{CI_i}{a_s + CI_i}\right),\qquad \text{Eq. S2}$$

where b_s is the survival probability without any competition and a_s is equal to the competition index the reduces the survival probability to 50% of b_s. Therefore, the effect of competition on survival increases with decreasing a_s.

Recruitment

New tree individuals, which are called “recruits” in the following, can either be offspring of trees in the local community or immigrants from the metacommunity. In spatially implicit neutral models the probability of immigration (m) has been usually treated as a free parameter that has to be estimated from data [3-5]. In contrast, in our modelling approach we link the probability of immigration to a spatially explicit kernel of recruitment distances as suggested by Chisholm & Lichstein [6]. According to their findings, the probability of immigration can be well approximated as:

$$m = \frac{P\, d_m}{\pi\, A}\qquad \text{Eq. S3}$$

where P is the perimeter of the plot (3000 m in this study), d_m is the mean distance between mother tree and recruit, and A is the plot area (50 ha).

Local Recruitment: With probability 1-m a recruit will be the offspring of one individual within the local community. Due to neutrality each tree in the local community has the same probability of being the ancestor of that recruit. To generate a recruit, we first randomly choose a mother tree, and second, we determine the location of the recruit, considering spatially-limited dispersal [7]. For this purpose, we use a radially-symmetric log-normal distance kernel [8,9] with the mean and the standard deviation of the distance between mother tree and recruit as parameters (d_m, d_{sd}). From the location of the mother tree we draw a uniformly distributed direction and a log-normally distributed distance, which together determine the position of the recruitment event.

We assumed periodic boundary conditions if the random location of the recruit was outside of the 50 ha plot, i.e. we treated the plot as a torus without edges [7,9] .

Immigration: In order to simulate non-spatial metacommunities, we apply the sequential construction scheme of Etienne & Alonso [5], which makes use of a simulation approach originally presented in Hubbell (2001), pg. 289. Thereby, the abundance distributions and thus the diversity of the metacommunity is determined by the fundamental biodiversity number θ, which is defined as $\theta = 2J_M\nu$, where J_M is the number of individuals in the metacommunity and ν is the speciation rate by point mutation (Hubbell 2001). We simulated metacommunities of 2×10^6 individuals, which represents ca. 100 times the size of the spatially-explicit local community.

With probability m a recruit in the local community will be the offspring of one individual within the local community (Hubbell 2001). According to neutrality each individual in the metacommunity has the same probability of being the ancestor of the immigrant and therefore the probability that the immigrant belongs to a certain species is equal to the relative abundance of that species in the metacommunity (Hubbell 2001). The recruit is position randomly within the simulated 50 ha plot.

Initialization and scheduling of model processes

In the beginning of each simulation a non-spatial metacommunity is generated. For the initial state in the local community 21100 recruits are drawn at random as immigrants from the metacommunity and randomly placed in the 50 ha plot. Then, we determine the competition indices and survival probabilities P_{surv} for all trees (Eqs. S1 and S2). Each model step includes the death of one tree, which is immediately replaced by a new recruit in order to fulfil the zero-sum assumption. To determine which tree dies, a random tree is selected and based on its P_{surv} it is randomly determined, if it dies or survives. If it survives a new tree is randomly tested for death or survival until a death event happens. As soon as a tree dies, a random recruitment event is simulated as explained above, either as immigration from the metacommunity or as local recruitment. The competition indices of the trees in the neighbourhoods of the dead tree and the new recruit are updated accordingly (Eqs. S1, S2). Each simulation proceeds for 100 generations. That means we iterated the model until the total number of death-recruitment events equals 100 times the number of trees in the local community. We graphically checked that this time was sufficient time to approach a dynamic equilibrium with regard to the investigated patterns (Fig. S2).

Selection of parameter ranges

We constrained the ZOI radius (r_t) to the range of 2 m – 6 m, as preliminary simulations indicated that values outside of this *a priori* range result in clearly unrealistic pattern with respect to tree spacing. In addition this restriction increased computational efficiency, because simulations with larger ZOI radii are computationally costly in evaluation of tree overlap. We could constrain the lower range of the 5-year survival probability without competition (b_s) to the observed mean inter-census survival rate of trees with dbh $\geq$ 10 cm in BCI (0.89). Considering the model definition (Eq. S2) we know *a priori* that lower values of b_s would result in an underestimation of tree survival and thus to an overestimation of the observed mortality rate. The ranges for the other four parameters represent low *a priori* information and result in large variation in the community patterns explained below.

Table S1. Parameters of the spatially explicit neutral model CONFETTI. Parameter values for the simulations were randomly drawn from the specified range assuming a uniform distribution.

**parameter**	**description**	***a priori* range**
r_t	radius of trees' zone-of-influence (ZOI)	2 m – 6 m
a_s	competition index that lowers survival probability by 50%	1 – 100
b_s	survival probability without competition	0.89 – 1.0
θ	fundamental biodiversity number of the metacommunity	10 – 100
d_m	mean recruitment distance	10 m – 100 m
d_{sd}	standard deviation of the recruitment distance	5 m – 50 m

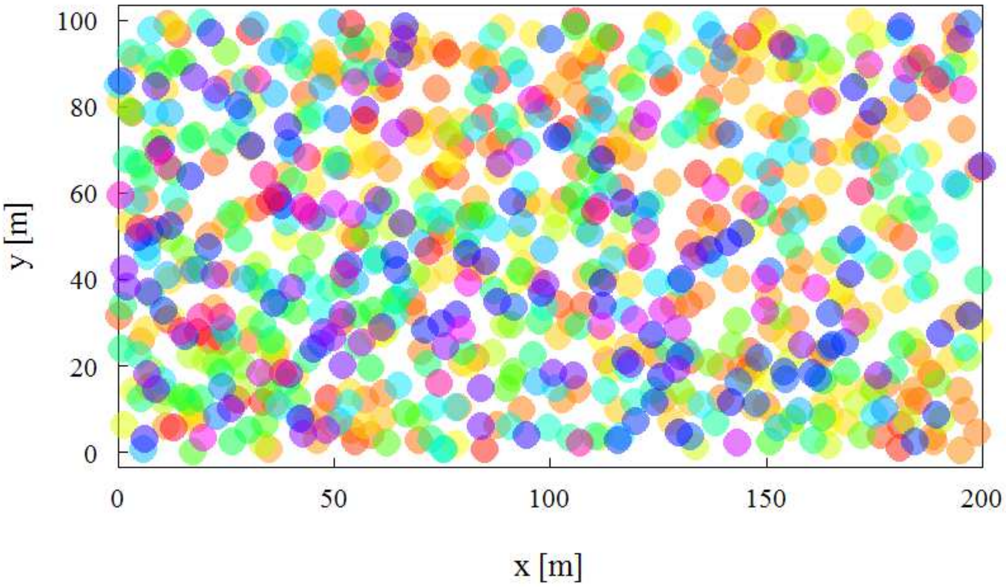

Figure S1. Snapshot of the spatially explicit simulation model. The panel represents a subplot of 200 m × 100 m. The circles represent tree's zones-of-influence (ZOI) where the size of the circles was scaled to the ZOI radius $r_t = 3.1$ m. Only trees with overlapping ZOIs compete with each other. The colour represents the species identity. In total there are 910 trees of 89 species in this subplot. The virtual census was sampled at the end of an example simulation run after tree 100 generations. As parameter values for the presented simulation we used the mean parameter values of the selection shown in Fig. S4: $r_t = 3.1$ m, $a_s = 17.4$, $b_s = 0.95$, $\theta = 55.8$, $d_m = 25.3$ m, $d_{sd} = 24.4$ m. This selection represented the best compromise in fitting all patterns simultaneously.

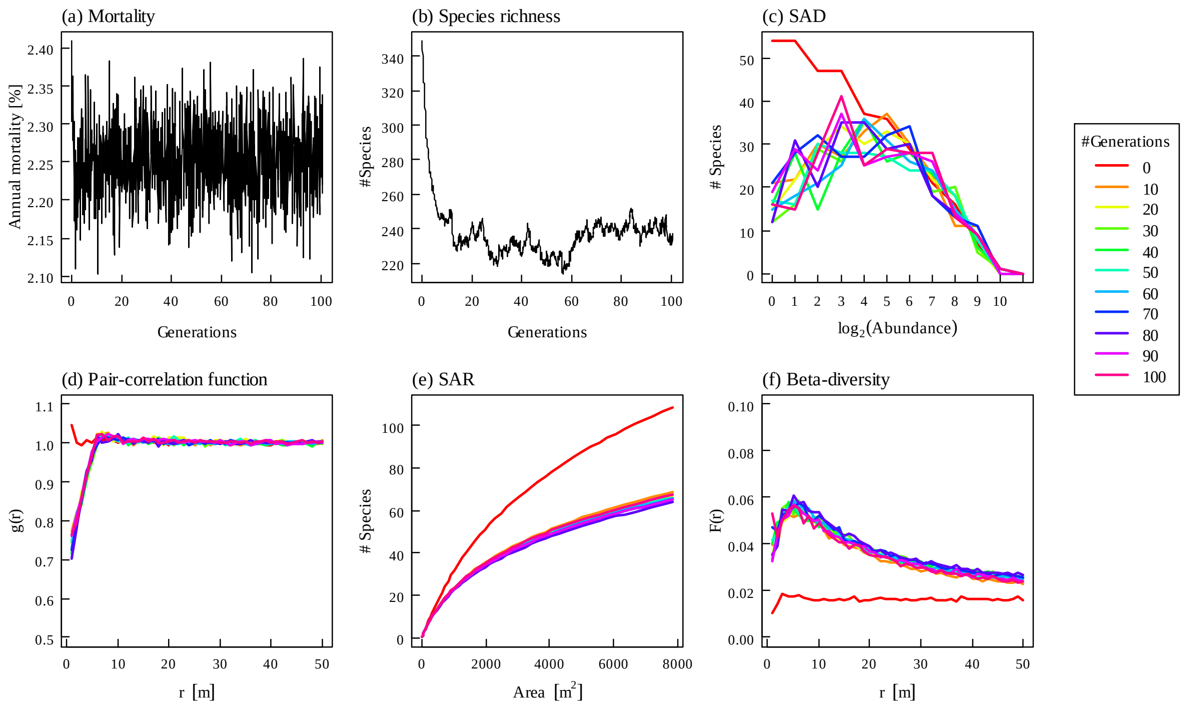

Figure S2. Temporal dynamics of an example simulation run of the spatially explicit model. For the mortality rate and the species richness the full time trajectory is shown (a, b). For the other patterns a snapshot was taken in the beginning and after each tenth generation. As parameter values for the presented simulation we used the mean parameter values of the selection shown in Fig. S4: $r_t = 3.1$ m, $a_s = 17.4$, $b_s = 0.95$, $\theta = 55.8$, $d_m = 25.3$ m, $d_{sd} = 24.4$ m. This selection represented the best compromise in fitting all patterns simultaneously.

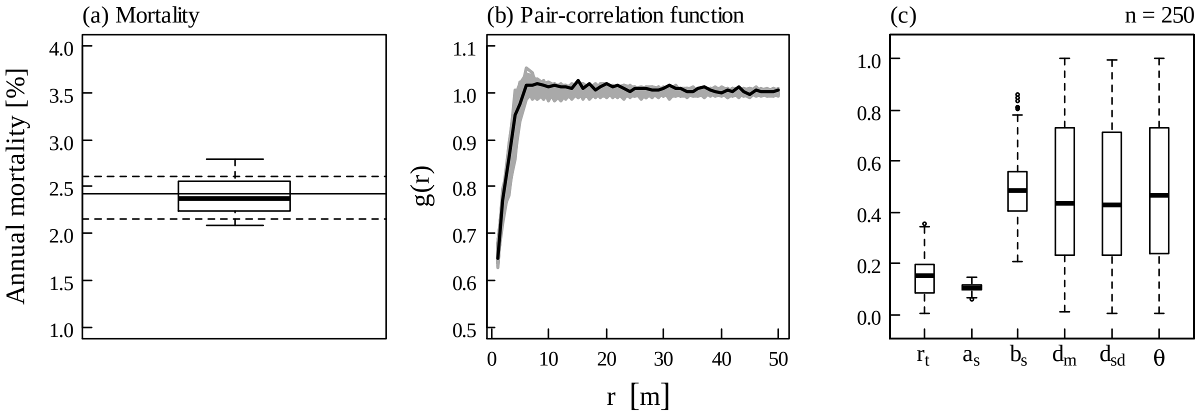

Figure S3. Model predictions and field observations when parameterizations were selected with the mortality rate and the pair-correlation function. For each pattern *i* the selection criterion was $mRD_i < \min(2\times\varepsilon_i, 0.2)$ (Table 1 in main text). Panels (a,b) show simulation results and observations, and the boxplots (c) summarize the selected parameter values and the number of selected parameter sets (*n*). For comparability the parameters were standardized to the range [0;1] (see Table S1). For the mortality rate (a) the boxplot on the left summarize the *n* simulation results, while the solid line and the dashed horizontal lines indicate the mean and the range in the five BCI censuses. For the pair-correlation function (b) the grey lines show the *n* simulation results and the black solid lines show the observed pattern averaged over the five BCI censuses.

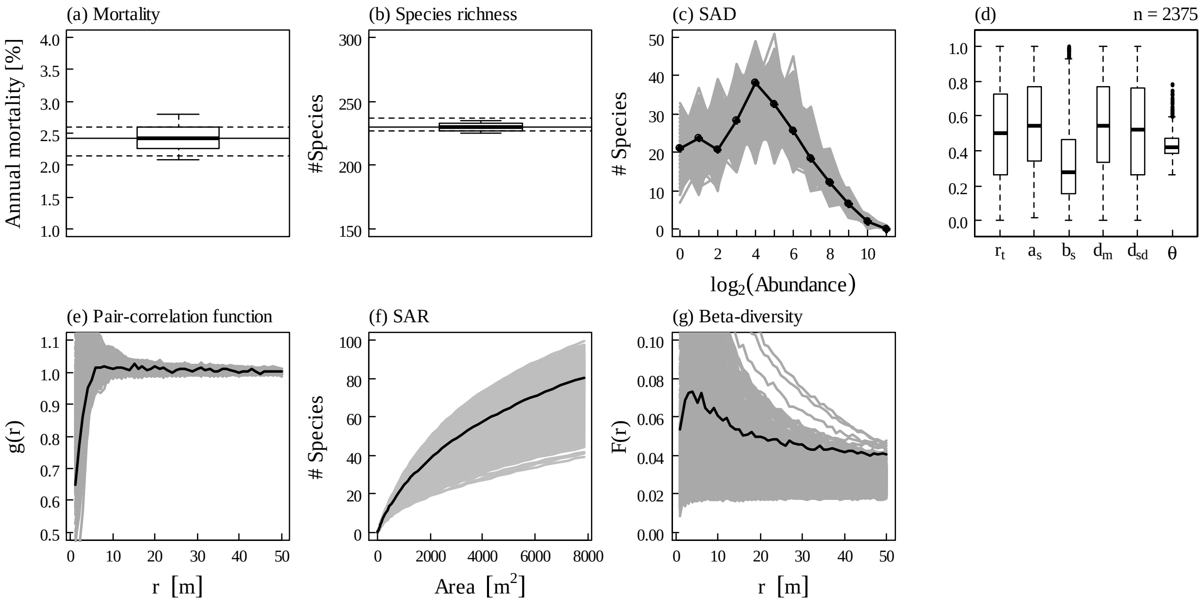

Figure S4. Model predictions and field observations when parameterizations were selected with the three non-spatial patterns mortality rate, species richness, and species abundance distributions (SAD). For each pattern *i* the selection criterion was $mRD_i < \min(2\times\varepsilon_i, 0.2)$ (see Table 1 in the main text). Panels (a-c, e-g) show simulation results and observations, and the boxplots (d) summarize the selected parameter values and the number of selected parameter sets (*n*). For comparability the parameters were standardized to the range [0;1] (see Table S1). For the scalar patterns – (a) mortality and (b) species richness – the boxplots on the left summarize the *n* simulation results, while the solid line and the dashed horizontal lines indicate the mean and the range in the five BCI censuses. For the species abundance distribution (c) and the spatial patterns (e)-(g), the grey lines show the *n* simulation results and the black solid lines show the observed pattern averaged over the five BCI censuses.

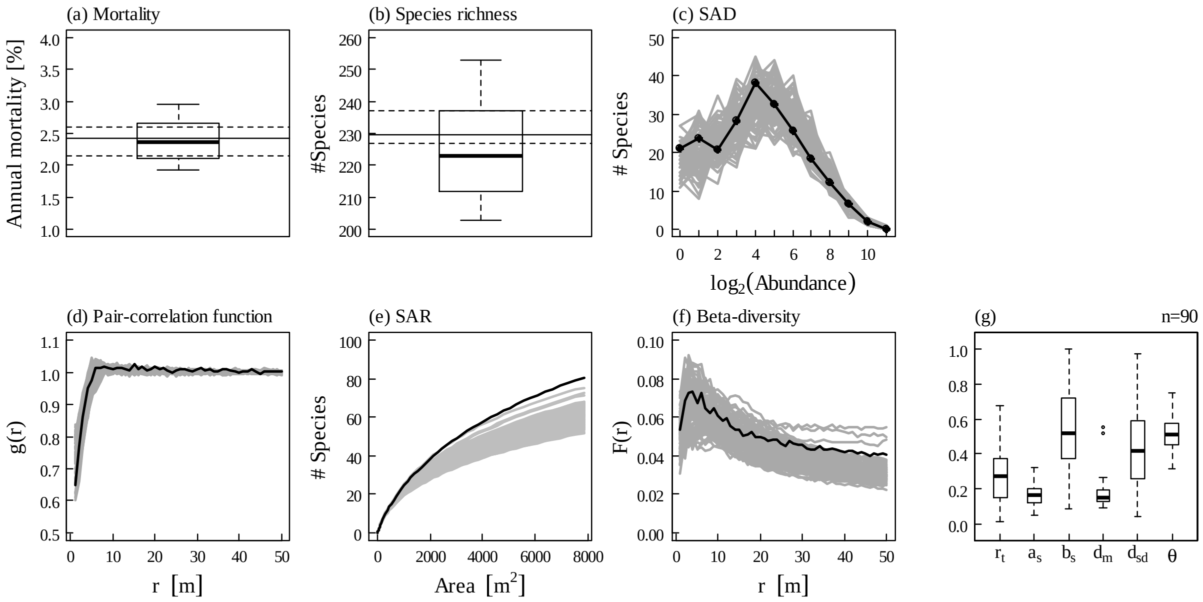

Figure S5. Model predictions and field observations when parameterizations were selected with all six patterns simultaneously. For each pattern *i* the selection criterion was $mRD_i < \min(10\times\varepsilon_{i,}\, 0.2)$ (Table 1). Panels (a-f) show simulation results and observations, and the boxplots (g) summarize the selected parameter values and the number of selected parameter sets (*n*). For comparability the parameters were standardized to the range [0;1] (see Table S1). For the scalar patterns – (a) mortality and (b) species richness – the boxplots on the left summarize the *n* simulation results, while the solid line and the dashed horizontal lines indicate the mean and the range in the five BCI censuses. For the species abundance distribution (c) and the spatial patterns (d)-(f), the grey lines show the *n* simulation results and the black solid lines show the observed pattern averaged over the five BCI censuses.

References

1. Weiner J, Stoll P, Muller-Landau H, and Jasentuliyana A. 2001 The effects of density, spatial pattern, and competitive symmetry on size variation in simulated plant populations. *Am Nat* **158**, 438-450. (doi:10.1086/321988)

2. May F, Grimm V, and Jeltsch F. 2009 Reversed effects of grazing on plant diversity: the role of below-ground competition and size symmetry. *Oikos* **118**, 1830-1843. (doi:10.1111/j.1600-0706.2009.17724.x)

3. Hubbell SP. 2001 *The unified neutral theory of biodiversity and biogeography,* 1 edn. Princeton, NJ: Princeton University Press.

4. Volkov I, Banavar JR, Hubbell SP, and Maritan A. 2003 Neutral theory and relative species abundance in ecology. *Nature* **424**, 1035-1037. (doi:10.1038/nature01883)

5. Etienne RS and Alonso D. 2005 A dispersal-limited sampling theory for species and alleles. *Ecol Lett* **8**, 1147-1156. (doi:10.1111/j.1461-0248.2005.00817.x)

6. Chisholm RA and Lichstein JW. 2009 Linking dispersal, immigration and scale in the neutral theory of biodiversity. *Ecol Lett* **12**, 1385-1393. (doi:10.1111/j.1461-0248.2009.01389.x)

7. Chave J, Muller-Landau HC, and Levin SA. 2002 Comparing classical community models: Theoretical consequences for patterns of diversity. *Am Nat* **159**, 1-23. (doi:10.1086/324112)

8. Greene DF, Canham CD, Coates KD, and Lepage PT. 2004 An evaluation of alternative dispersal functions for trees. *J Ecol* **92**, 758-766. (doi:10.1111/j.0022-0477.2004.00921.x)

9. May F, Giladi I, Ziv Y, and Jeltsch F. 2012 Dispersal and diversity - unifying scale-dependent relationships within the neutral theory. *Oikos* **121**, 942-951. (doi:10.1111/j.1600-0706.2011.20078.x)
